# WNT Signaling Pathway Gene Polymorphisms and Risk of Hepatic Fibrosis and Inflammation in HCV-Infected Patients

**DOI:** 10.1371/journal.pone.0084407

**Published:** 2013-12-30

**Authors:** Yanhong Liu, Hashem B. El-Serag, Li Jiao, JuSeog Lee, David Moore, Luis M. Franco, Shahriar Tavakoli-Tabasi, Spiridon Tsavachidis, Jill Kuzniarek, David J. Ramsey, Donna L. White

**Affiliations:** 1 Department of Pediatrics, Baylor College of Medicine, Houston, Texas, United States of America; 2 Section of Gastroenterology and Hepatology, Michael E. DeBakey VA Medical Center and Baylor College of Medicine, Houston, Texas, United States of America; 3 Clinical Epidemiology and Comparative Effectiveness Program, Section of Health Services Research (IQuEST), Michael E. DeBakey VA Medical Center and Baylor College of Medicine, Houston, Texas, United States of America; 4 Dan L. Duncan Cancer Center at Baylor College of Medicine, Houston, Texas, United States of America; 5 Texas Medical Center Digestive Disease Center, Houston, Texas, United States of America; 6 Center for Translational Research on Inflammatory Diseases (CTRID), Michael E. DeBakey Veterans Affairs Medical Center, Houston, Texas, United States of America; 7 Department of Systems Biology, MD Anderson Cancer Center, Houston, Texas, United States of America; 8 Department Molecular and Cell Biology and Department of Medicine, Baylor College of Medicine, Houston, Texas, United States of America; 9 Department of Molecular and Human Genetics, Baylor College of Medicine, Houston, Texas, United States of America; 10 Section of Infectious Diseases, Michael E. DeBakey Veterans Affairs Medical Center and Baylor College of Medicine, Houston, Texas, United States of America; Saint Louis University, United States of America

## Abstract

**Background:**

Chronic hepatitis C infection is the leading cause of hepatocellular carcinoma (HCC), a highly lethal malignancy with rapidly increasing prevalence in the United States. Little is known about genetic variations and HCC risk. This study aimed to determine if genetic variation in Wnt signaling pathway genes are associated with advanced hepatic fibrosis and inflammation risk in a hepatitis C virus (HCV) infected population.

**Methods:**

We performed a genetic association cross-sectional study evaluating single nucleotide polymorphisms (SNPs) in 58 candidate genes and risk of FibroSURE-Acti Test determined advanced fibrosis (F3/F4-F4 advanced cases vs. F0-F3 mild controls) and inflammation (A2/A3-A3 advanced cases vs. A0-A2 mild controls). We calculated odds ratios (ORs) and 95% confidence intervals (CIs) employing multivariate logistic regression. Haplotypes were inferred by the HAPLO.STAT program, interactions were evaluated using multifactor dimensionality reduction (MDR) analysis.

**Results:**

Among 425 chronically HCV-infected male veterans, 155 (37%) had advanced fibrosis and 180 (42%) had advanced inflammation. Of 3016 SNPs evaluated, eight were significantly associated with fibrosis risk (e.g., *SFRP2* rs11937424: OR = 2.19, 95% CI 1.48-3.23, *P* = 0.00004), and seven were significantly associated with inflammation risk (e.g., *SFRP1* rs16890282: OR = 2.15, 95% CI 1.39-3.16, *P* = 0.0004). MDR analysis identified overweight/obese, S*OST* rs1405952, *SFRP2* rs11937424, and *FZD4* rs11234870 as the best interaction model for predicting risk of fibrosis; whereas race/ethnicity, *FZD1* rs1346665, and *TBX3* rs1520177 as the best interaction model for predicting risk of inflammation.

**Conclusions:**

Polymorphisms in several genes involved in the Wnt signaling pathway were associated with hepatic fibrosis or inflammation risk in HCV-infected males. Additional studies in other multi-ethnic HCV cohorts are needed to validate our findings in males and to assess if similar associations exist in chronically HCV-infected females.

## Introduction

The World Health Organization (WHO) estimates that globally from 2-3% of the world’s population (2-3%) are infected with the hepatitis C virus (HCV)[1]. In the United States, approximately 3.9 million people in the United States have been infected with HCV, of whom 2.7 million are chronically infected [2]. HCV infection progresses over a variable time period of 20 to 40 years through several stages of hepatic fibrosis that culminates in cirrhosis in up to one-third of chronic HCV patients. Advanced hepatic fibrosis including cirrhosis is the primary precursor lesions for most cases of hepatocellular carcinoma (HCC). The incidence of HCC in the United States has more than tripled since the mid-1980s, with most of this increase attributed to HCV-related liver disease [3]. Finding risk factors for the progression of HCV-related liver disease could have significant clinical implications (i.e., detection and prognosis) and public health implications. Several environmental (e.g., alcohol) as well as host factors (e.g., older age, male sex, and diabetes) have been shown to influence the risk of developing advanced fibrosis or cirrhosis. However, much less is known about host genetic factors. 

The Wnt signaling pathway is gaining prominence given its critical roles in many facets of human biology, particularly in embryonic development as well as maintaining tissue and organ homeostasis. Recent studies have described the importance of Wnt signaling in regulating liver cell proliferation during development [4,5] and in governing essential functions in the adult liver [6,7]. HCV infection has been associated increased expression of Wnt and several upstream and downstream genes in its biochemical pathways [8], with a recent gene expression study in 61 HCV patients in Japan showing changes in several WNT pathway genes with moderate (F2/F3) fibrosis levels [9]. Moreover, somatic mutations leading to aberrant activation of the Wnt signaling pathway genes have been found in several types of cancer including HCC and hepatoblastoma [10,11]. 

Genome structure variations including single nucleotide polymorphism (SNP) have been identified for genes coding Wnt and its related signaling pathway and are likely to play a role in inter-individual variability of expression of target genes downstream in the pathway. However, there are no epidemiologic genetic association studies of Wnt signaling in advanced hepatic fibrosis or cirrhosis in HCV infected individuals. Hence, the primary aim of our study was to test the hypothesis that host germline genetic variation in Wnt signaling pathway genes is associated with increased hepatic fibrosis and inflammation in a well-characterized HCV-infected male population. 

## Methods

### Study Population and Design

We prospectively recruited consecutive HCV-infected veterans eligible for study participation at the time of their appointment at a HCV clinic between May, 2009 and December, 2012. Details of the study were previously described [12]. Briefly, prior to the clinic visit, veterans completed a research-assistant administered survey that interrogated medical and risk factor history including lifetime alcohol use, had a fasting venipuncture performed for standard clinical laboratory tests and to perform the FibroSure-ActiTest as a measure of hepatic pathology, and had anthropometric measurements taken. We restricted our current cross-sectional analysis to: 1) Caucasian and African American male veterans aged 18-70 years (who collectively represent 90%+ of HCV+ veterans in the target population); 2) who had no electronic medical record (EMR) or self-reported history of liver transplant, decompensated liver disease, HCC, dementia, or psychosis; 3) were serologically-confirmed at enrollment to have HCV viremia and to be negative for HIV or active HBV infection; 4) were not on HCV antiviral therapy at study recruitment; and 5) who had completed germline DNA genotyping and FibroSURE testing as of February 2013. This research was approved by the Institutional Review Boards for the Michael E. DeBakey VA Medical Center and Baylor College of Medicine (IRB protocol number H-22934), with RAs obtaining signed witnessed informed consents from study participants using the dual IRB-approved consent form. This study was funded in part by the Department of Veteran Affairs, the National Institute of Diabetes Digestive and Kidney Diseases, and the Diana Helis Henry Medical Research Foundation. The funders played no role in study design, data collection and analysis, decision to publish, or preparation of the manuscript.

### Study Measures

#### Outcome and Confounder Variables

The primary outcome (hepatic disease severity) variables were FibroSURE-ActiTest determined hepatic fibrosis and inflammation. FibroSURE (also known as FibroTest, BioPredictive, France) uses a proprietary algorithm incorporating serum levels of α2-macroglobulin, apolipoprotein A1, haptoglobin, total bilirubin, and γ-glutamyl-transpeptidase to determine fibrosis level and adds ALT to determine necroinflammatory activity level. These scores are categorized into METAVIR biopsy-based equivalent degrees of hepatic fibrosis. FibroSURE has demonstrated a high concordance with biopsy-assessed level of METAVIR pathology in HCV-infected populations [13-17]. We defined advanced fibrosis cases as individuals with FibroSURE-determined fibrosis stage F3/F4 thru F4, cirrhosis, and mild fibrosis controls as individuals with stages F0-F3, and advanced inflammatory activity cases individuals with inflammatory grades A2/A3-A3 and mild activity controls as A0-A2. We measured several potential confounders for advanced liver disease including adiposity, diabetes, and alcohol abuse. Weight in pounds was measured using the InBody520 Direct Segmental 8-point Multi-frequency Bioelectrical Impedance Analysis scale, with height in inches obtained using a study designated stadiometer. Body mass index (BMI) was calculated using the Quetelet index formula, with overweight/obesity defined as a BMI of 25 or higher. Diabetes was defined as present based on positive history from either patient self-report or EMR review or if fasting glucose was >120 absent a positive history of diabetes. Chronic alcohol abuse was defined as self-reported history of consuming 3 or more drinks a day for at least 10 consecutive years. 

#### Selection of Genes, SNPs and Genotyping Assays

We identified a set of 58 candidate genes involved in the Wnt signaling pathway, including Wnt ligands, frizzled receptors, TCF factors, Dishevelled, SFRP family, and DKK members (for full gene list, please see Table S1). A total of 3454 SNPs were identified from Illumina HumanOmni 2.5-8 microarrays (Illumina, San Diego, CA). After excluding the monomorphic SNPs and SNPs with minor allele frequency (MAF) lower than 0.05, the vast majority of our final 3016 SNPs were located in flanking and intronic regions.

Sample preparation and array staining were performed in blinded manner in batches with randomly selected cases and controls in accordance to Illumina protocols at the Baylor College of Medicine (BCM) Laboratory for Translational Genomics. Arrays were scanned on an Illumina iScan system. SNP clustering and basic quality control of the genotyping data was performed on GenomeStudio software, version 2010 (Illumina, Inc., San Diego, CA, U.S.A.). All microarrays had call rates >0.99. To evaluate the possibility of undisclosed familial relationships or duplicated samples, we performed identity-by-state (IBS) clustering on the basis of autosomal genotypes, followed by multidimensional scaling analysis of the resulting matrix of IBS pairwise distances using PLINK. This quality control step identified no samples from related or duplicated individuals. 

### Statistical Methods

We assessed goodness of fit to the Hardy-Weinberg equilibrium (HWE) expectation for each SNP using a χ^2^ test. Genotype frequencies of advanced and mild subjects were compared using χ^2^ tests. We employed unconditional logistic regression analysis to evaluate the association between individual Wnt signaling pathway SNPs and risk of advanced hepatic fibrosis (advanced F3/F4-F4 vs. mild F0-F3 fibrosis) and also of advanced hepatic inflammation (advanced A2/A3-A3 vs. mild A0-A2 inflammation). The Akaike's information criterion was used to determine the genetic model for each SNP [18]. Odds ratios (ORs) and 95% confidence intervals (CIs) were calculated in multivariate analysis after adjusting for age, race/ethnicity, overweight/obesity (BMI≥25), diabetes, chronic alcohol abuse, viral load, and HCV genotypes. We also compared our race-adjusted models to comparable race-stratified models to assess adequacy of race-adjustment. To reduce the potential of spurious findings due to multiple testing, we applied 10,000 permutations to empirically derived allelic association *P* values using a two-sided *P*-value of 0.001 to assess significance. To further evaluate the chance of obtaining a false-positive association in our dataset, we also used the false-positive report probability (FPRP) test using the moderate range of prior probabilities of 0.05 - 0.01 and FPRP cutoff value 0.2 [19]. Next, we performed a joint effect analysis by adding up the number of adverse alleles of the significant SNPs identified from the main effects analysis. Adverse alleles were defined as the minor allele of the risk-affect SNPs (OR > 1) and the common allele of the protective-effect SNPs (OR < 1). 

For detecting and characterizing nonlinear interactions among SNP-SNP and SNP-environment risk factors (e.g., age and diabetes), we used the multifactor dimensionality reduction (MDR) method [20-22]. The nonparametric MDR combines attribute selection and classification with cross-validation and permutation testing to provide a powerful approach to detecting nonlinear interactions. The model with the highest testing accuracy and the highest cross-validation consistency (CVC) was selected and further evaluated using the permutation test performed by shuffling groups with mild and advanced fibrosis or inflammation 10,000 times and repeating the MDR analysis on each randomized dataset. MDR was performed using MDR version 2.0 beta 8.4 that is freely available online (http://www.epistasis.org/software.html). 

We similarly evaluated the association between haplotypes for each gene with more than 1 SNP in strong linkage disequilibrium (LD) that was significantly associated with increased risk of advanced fibrosis or inflammation in single locus analysis. Pairwise LD among SNPs was examined using Lewontin’s standardized coefficient *D’* and LD coefficient *r* ^*2*^ [23], with “strong LD” defined as the one-sided upper 95% CI boundary on *D’* is > 0.98 and the lower boundary is > 0.7. We performed our haplotype analysis using the HAPLO.STATS package developed by Schaid et al. (http://www.mayo.edu/hsr/Sfunc.html) as implemented in R [24]. Haplotypes with a frequency of less than 0.03 were pooled into a combined group and empirical *P* values based on 10, 000 simulations were computed for all tests.

## Results

### Characteristics of the study population

Table 1 summarizes the characteristics of the 425 HCV-infected male patients included in our analysis. The mean age of our HCV cases was 59 years (range 18-70), and the majority (74%) was of White non-Hispanic race/ethnicity. There were 270 (64%) with mild fibrosis (stage ≤F3) and 155 (36%) with advanced fibrosis (stage > F3), and 245 (58%) with mild inflammation (grade ≤A2) and 180 (42%) with advanced inflammation (grade > A2) based on FibroSURE testing. Compared to HCV+ males with mild fibrosis or inflammation, those with advanced fibrosis or inflammation were more likely to be overweight/obese (*P* = 0.02 and 0.01, respectively) and to be White (*P* = 0.09 and 0.004, respectively). There were no differences in the prevalence of HCV genotype, viral load, chronic alcohol abuse, or diabetes among patients with mild and advanced fibrosis or inflammation. 

**Table 1:** Sociodemographic and clinical characteristics of male veterans with chronic hepatic C virus (HCV) infection based on FibroSURE-determined hepatic fibrosis and inflammation.

		**Advanced hepatic fibrosis risk**					**Advanced hepatic inflammation risk**			
Risk Factor	All, N=425	Mild fibrosis	Advanced fibrosis	OR (95%	P-value		Mild inflammation	Advanced inflammation	OR (95%	P-value
	(%)	(F0-F3)	(F3/F4-F4)	CI)			(A0-A2)	(A2/A3-A3)	CI)	
		n = 270 (%)	n = 155 (%)				n = 245 (%)	n = 180 (%)		
Race/Ethnicity					0.0907					0.0035
White	313 (74)	191 (71)	122 (79)	1			167 (68)	146 (81)	1	
Black	112 (26)	79 (29)	33 (21)	0.65 (0.41-1.04)			78 (32)	34 (19)	0.49 (0.31-0.78)	
Age-group					0.0926					0.1657
≤55	183 (43)	125 (46)	58 (37)	1			98 (40)	85 (47)	1	
>55	242 (57)	145 (54)	97 (63)	1.44 (0.96-2.15)			147 (60)	95 (53)	0.7451 (0.51-1.09)	
Chronic alcohol abuse***^^^***,***^&^***					0.9754					0.7510
Negative	162 (38)	102 (38)	60 (39)	1			91 (37)	71 (37)	1	
Positive	261 (62)	166 (62)	95 (61)	0.97 (0.65-1.46)			152 (63)	109 (61)	0.91 (0.62-1.36)	
Diabetes**^*^*^**					0.2659					0.8280
Negative	325 (77)	212 (79)	113 (74)	1			189 (78)	136 (76)	1	
Positive	96 (23)	56 (21)	40 (26)	1.34 (0.84-2.12)			54 (22)	42 (24)	1.08 (0.68-1.71)	
Overweight/obese***^^^***,***^#^***					0.0239					0.0141
Negative	104 (25)	76 (28)	28 (18)	1			71 (29)	33 (18)	1	
Positive	320 (75)	193 (72)	127 (82)	1.79 (1.10-2.91)			173 (71)	147 (82)	1.82 (1.15-2.92)	
Viral Load					0.208					0.234
Low	139 (33)	91 (34)	48 (31)	1			78 (32)	61 (34)	1	
Medium	140 (33)	81 (30)	59 (38)	1.4 ( 0.83 - 2.31 )			75 (31)	65 (36)	1.1 ( 0.67 - 1.83 )	
High	139 (33)	94 (35)	45 (29)	0.91 ( 0.53 - 1.54 )			88 (36)	51 (28)	0.74 ( 0.45 - 1.23 )	
HCV genotyping					0.798					0.079
1	338 (80)	214 (79)	124 (80)	1			202 (82)	136 (76)	1	
2 and 3	80 (19)	52 (19)	28 (18)	0.93 ( 0.54 - 1.59 )			39 (16)	41 (23)	1.6 ( 0.93 - 2.63 )	

^^^ Numbers do not add up to the column totals due to missing values.

^&^ Defined as 3+ drinks/day for at least 10 consecutive years.

^#^ Overweight/obesity (BMI >=25).

### Association between individual SNP and advanced hepatic fibrosis or inflammation risk

Of the 3016 SNPs analyzed in single locus analyses, there were 35 SNPs associated with advanced fibrosis risk and 48 SNPs associated with advanced inflammation risk at the P ≤ 0.01 (Table S2). At the P ≤ 0.001 level, eight SNPs were significantly associated with advanced fibrosis risk*: SFRP2* rs11937424, *SFRP2* rs7673508*, SFRP2* rs6853435, *SFRP2* rs3810765, *FZD4* rs11234870, *SOST* rs1405952, *CTNNB1* rs1798796 and *CSNK1A1* rs2431718; and seven were significantly associated with advanced inflammation risk: *FZD1* rs1476442 and rs1346665, *SFRP1* rs16890282, *FZD8* rs3904594 and rs7920455, *TBX3* rs1386037 and rs1520177. The genotype distributions of SNPs associated with either advanced fibrosis (F3/F4-F4) or advanced inflammation (A2/A3-A3) at the P ≤ 0.001 are summarized in Table 2. In multivariate logistic regression analysis, the strongest signal for advanced fibrosis risk was seen with *SFRP2* rs11937424 (OR = 2.19; 95% CI 1.48-3.23; *P* = 0.00004) and for advanced inflammation risk was seen with *SFRP1* rs16890282 (OR = 2.15; 95% CI 1.39-3.16; *P* = 0.0004). 

**Table 2:** Genotype frequencies of SNPs and the associations with advanced hepatic fibrosis or inflammation risk in patients with active HCV infection (P ≤ 0.001).

					MAF						Logistic regression*			FPRP Prior ^$^	
Chr	SNP ID **^*ǂ*^**	Gene	Position	Alleles	Advanced group	Mild group	Allelic *P*-value^&^		Allelic Permutation *P*-value^#^		OR (95% CI)	*P*-value		*0.05*	*0.01*
**Advanced hepatic fibrosis risk**					F3/F4-F4	F0-F3									
3	rs1798796 **^*r*^**	*CTNNB1*	41190647	G/A	0.15	0.22	0.005		0.097		0.20 (0.07-0.62)	0.0003		0.761	0.943
4	rs6853435 **^*r*^**	*SFRP2*	154706016	A/G	0.39	0.49	0.001		0.027		0.65 (0.49-0.87)	0.0007		0.050	0.215
4	rs3810765 **^*r*^**	*SFRP2*	154709480	A/G	0.39	0.50	0.002		0.035		0.66 (0.50-0.89)	0.0008		0.059	0.245
4	rs11937424 **^*d*^**	*SFRP2*	154711186	G/A	0.35	0.23	0.0001		0.003		2.19 (1.48-3.23)	0.00004		0.037	0.168
4	rs7673508 **^*d*^**	*SFRP2*	154769597	G/A	0.46	0.34	0.0004		0.011		1.56 (1.14-2.13)	0.0006		0.057	0.239
5	rs2431718 **^*d*^**	*CSNK1A1*	148853121	G/A	0.34	0.45	0.001		0.028		0.49 (0.32-0.75)	0.0003		0.124	0.425
11	rs11234870 **^*d*^**	*FZD4*	86616127	C/A	0.32	0.22	0.001		0.025		2.10 (1.38-3.19)	0.0003		0.117	0.407
17	rs1405952 **^*d*^**	*SOST*	41753789	A/G	0.26	0.16	0.0005		0.012		2.35 (1.53-3.59)	0.00008		0.073	0.291
**Advanced hepatic inflammation risk**					A2/A3-A3	A0-A2									
7	rs1346665 **^*r*^**	*FZD1*	90919567	C/A	0.42	0.50	0.0146		0.175		0.40 (0.24-0.68)	0.0004		0.270	0.658
7	rs1476442 **^*d*^**	*FZD1*	91272344	A/C	0.27	0.24	0.2614		0.659		2.10 (1.35-3.22)	0.001		0.200	0.565
8	rs16890282 **^*d*^**	*SFRP1*	40949654	C/A	0.23	0.14	0.0003		0.004		2.15 (1.39-3.16)	0.0004		0.098	0.362
10	rs3904594 **^*r*^**	*FZD8*	36322229	A/G	0.45	0.41	0.0007		0.009		2.19 (1.43-3.31)	0.0005		0.137	0.453
10	rs7920455 **^*d*^**	*FZD8*	36345362	A/G	0.27	0.37	0.0018		0.028		0.53 (0.35-0.79)	0.001		0.164	0.505
12	rs1386037 **^*d*^**	*TBX3*	116139373	G/A	0.28	0.18	0.0006		0.007		1.64 (1.17-2.30)	0.0009		0.093	0.349
12	rs1520177 **^*d*^**	*TBX3*	116230118	A/G	0.41	0.50	0.018		0.213		0.48 (0.31-0.75)	0.001		0.225	0.602

NOTE: MAF, minor allele frequency; OR, odds ratio; CI, confidence interval; FPRP, False positive report probability; BFDP, Bayesian false-discovery probability.

^ǂ^ The Akaike's information criterion was used to determine the genetic model for each SNP. D, dominant; R, recessive.

^&^ *P* value for difference in allele distributions between patients with mild (≤ F3, ≤ A2) vs. advanced (F3, A2) hepatic fibrosis or inflammation.

^#^ Generated by permutation test with 10,000 times.

* Adjusted for age, race/ethnicity, presence of overweight/obesity (BMI > 25), chronic alcohol abuse, diabetes, viral load, and HCV genotypes.

^$^ Bold have a FPRP < 0.2 values at OR 1.5 level suggesting a true association.

To further evaluate the robustness of these findings, we calculated FPRP value at two levels of prior probabilities (0.05 and 0.01) for the eight significantly fibrosis associated SNPs and seven significantly inflammation associated SNPs (Table 2). At a prior probability level of 0.05, seven of these eight fibrosis associated SNPs and five of the seven inflammation associated SNPs remained noteworthy (FPRP ≤ 0.2, prior 0.05). At a very low prior probability of 0.01, only the fibrosis associated *SFRP2* rs11937424 SNP remained noteworthy (FPRP ≤ 0.2, prior 0.01). 

Race/ethnicity stratified analysis revealed that all SNPs identified as significant in race-adjusted analyses remained highly significant in non-Hispanic Whites, though the confidence intervals grew wider because of decreased sample size (Table S3) However, none of the SNPs remained significant in African Americans, who collectively represented <30% of all study participants.. We similarly stratified cases and controls by age (young ≤55 years vs. old >55 years), but observed no significant among age-groups (data not shown)

### Association between the cumulative effects of the SNPs and advanced hepatic fibrosis or inflammation risk

We next assessed the cumulative effects of the significant main effect SNPs (eight for fibrosis and seven for inflammation) in a joint analysis. We treated the minor allele of the risk-effect SNPs and the common allele of the protective-effect SNPs as adverse alleles. As shown in Table 3, a strong gene-dosage effect on risk for advanced fibrosis and advanced inflammation was observed when the SNPs were analyzed in combination (*P* trend < 0.0000001 for advanced fibrosis and advanced inflammation risk, respectively). 

**Table 3:** Cumulative genetic risk analysis of adverse genotypes on advanced hepatic fibrosis or inflammation risk in patients with HCV.

**No. of adverse genotypes** ^#^	**Advanced group (%)**	**Mild group (%)**	Logistic regression*		***P* trend**
			OR (95% CI)	*P*-value	
**Advanced hepatic fibrosis risk (F3/F4-F4 vs. F0-F3)**					< 0.0000001
	**F3/F4-F4 (%)**	**F0-F3 (%)**			
0-2	12 (7.74)	81 (30)	1		
3	25 (16.13)	83 (30.74)	2.06 (1.01-4.27)	0.042	
4	53 (34.19)	81 (30)	4.38 (2.23-8.77)	0.00001	
5-8	65 (41.93)	25 (9.26)	16.36 (8.18-32.5)	< 0.0000001	
**Advanced hepatic inflammation risk (A2/A3-A3 vs. A0-A2)**					<0.0000001
	**A2/A3-A3 (%)**	**A0-A2 (%)**			
0-1	24 (13.33)	100 (40.82)	1		
2	35 (19.44)	68 (27.76)	2.15 (1.19-3.88)	0.013	
3	44 (24.45)	41 (16.73)	4.48 (2.47-8.26)	0.000001	
4-7	77 (42.77)	36 (14.69)	8.91 (4.96-16.15)	< 0.0000001	

Note: OR, odds ratio; CI, confidence interval.

^#^ The cumulative genetic risk analysis was evaluated by adding up the number of adverse alleles of the significant SNPs identified from the main effects analysis. Adverse alleles were defined as the minor allele of the risk SNPs (fibrosis: *SFRP2* rs11937424, *SFRP2* rs7673508, *FZD4* rs11234870, *SOST* rs1405952; inflammation: *FZD1* rs1476442, *SFRP1* rs16890282, *FZD8* rs3904594, *TBX3* rs1386037), and the common allele of the protective SNPs (fibrosis: *CTNNB1* rs1798796, *SFRP2* rs6853435, *SFRP2* rs3810765, *CSNK1A1* rs2431718; inflammation: *FZD1* rs1346665, *FZD8* rs7920455, *TBX3* rs1520177)

* Adjusted for age, race/ethnicity, presence of overweight/obesity (BMI>25), chronic alcohol abuse, diabetes, viral load, and HCV genotypes.

### Association between haplotypes and advanced hepatic fibrosis or inflammation risk

Because four *SFRP2 SNPs* showed strong association with advanced fibrosis risk, we conducted haplotype analysis for *SFRP2*. These four SNPs were in strong LD with each other (D’ > 0.7, see Table S4). Table 4 summarizes the haplotype frequencies and associations with mild and advanced fibrosis or inflammation. Consistent with the individual SNP analyses, of the four observed haplotypes, risk haplotypes “A A A A” with carriage of all four adverse alleles of the four SNPs conferred the strongest haplotype association (adjusted OR = 1.93, 95% CI 1.36 - 2.72; *P* = 0.0003). Haplotype analysis of *FZD1*, *FZD8* and *TBX3* also identified significant associations between their respective individual haplotype profiles and advanced inflammation risk (global *P* values = 0.029, 0.0035, and 0.0008, respectively). 

**Table 4:** Haplotype analyses and associations with advanced hepatic fibrosis or inflammation risk in patients with HCV infection.

Gene	Blocks Haplotype**^*&*^**	Haplotype frequency			Logistic regression*		Global *P * **^*#*^**
		Total	Mild group	Advanced group	OR (95% CI)	*P*-value	
**Advanced hepatic fibrosis risk**							
Advanced Fibrosis risk							
*SFRP2*			**F3/F4-F4**	**F0-F3**			
	G G G G	0.45	0.49	0.39	1		0.0018
		0.225					
		0.1475					
		0.1211					
		0.044					
		0.0102					
	A A A A	0.23	0.19	0.29	1.93 (1.36-2.72)	0.0003	
	A A G A	0.15	0.13	0.17	1.59 (1.07-2.16)	0.015	
	A A G G	0.12	0.15	0.09	0.97 (0.66-1.42)	0.23	
	Rare	0.05	0.04	0.06	1.75 (0.95-3.31)	0.1	
**Advanced hepatic inflammation risk**							
*FZD1*			**A2/A3-A3**	**A0-A2**			
	C A	0.42	0.40	0.44	1		0.029
	A A	0.33	0.36	0.30	0.82 (0.57 - 1.17)	0.27	
	A C	0.13	0.14	0.11	0.73 (0.46 - 1.16)	0.18	
	C C	0.12	0.10	0.15	1.60 (0.95 - 2.70)	0.081	
*FZD8*							
	A G	0.48	0.43	0.55	1		0.0035
	G A	0.33	0.37	0.26	0.59 (0.43 - 0.80)	0.0004	
	A A	0.19	0.19	0.18	0.75 (0.52 - 1.09)	0.13	
	G G	0.01	0	0.01	NA**^*ǂ*^**	<0.0001	
*TBX3*							
	A G	0.41	0.41	0.42	1		0.0008
	G G	0.37	0.41	0.30	0.68 (0.48-0.96) 2.11)	0.028	
	A A	0.13	0.10	0.16	1.56 (0.97-2.51) 1.08)	0.063	
	G A	0.09	0.08	0.12	1.35 (0.77-2.31)	0.29	

NOTE: OR, odds ratio; CI, confidence interval; N.A, not available.

Loci chosen for fibrosis risk and SFRP2 haplotype analysis: rs6853435, rs3810765, rs11937424, rs7673508; Loci chosen for inflammation risk analysis: *FZD1* (rs1346665, rs1476442), *FZD8* (rs7920455, rs3904594), and *TBX3* (rs1386037, rs1520177).

^*^ Adjusted for age, race/ethnicity, presence of overweight/obesity (BMI > 25), chronic alcohol abuse, diabetes, viral load, and HCV genotypes.

^#^ Generated by permutation test with 10,000 times.

^&^ Haplotypes with a frequency less than 0.05 were pooled into one mixed group.

^ǂ^ Given rarity of haplotype, haplotype not evaluated in statistical tests of association.

### Gene-gene and gene-environment interactions

The clinical variables (race/ethnicity, age, chronic alcohol abuse, diabetes, overweight/obesity, viral load, and HCV genotypes) and the noteworthy SNPs from the main effect analysis (eight for fibrosis and seven for inflammation) were included in the MDR analysis. Table 5 summarizes the best interaction models obtained in the MDR analysis for advanced fibrosis and for advanced inflammation risk. Firstly, in models for fibrosis risk association, *SFRP2* rs11937424 was the best one-locus model for predicting advanced fibrosis risk, which confirmed the main effect of the *SFRP2*; but among these five models, the best interaction model was a 4-locus (i.e., model with overweight/obese, *SOST* rs1405952, *SFRP2* rs11937424 and *FZD4* rs11234870), with an improved testing accuracy to 0.68 (CVC = 97, *P* < 0.0001). Secondly, in models for inflammation risk association, *SFRP1* rs16890282 was the best one-locus model. Of these five candidate interaction models, the 3-locus model (i.e., race/ethnicity, *FZD1* rs1346665, and *TBX3* rs1520177) was the best; it had a perfect CVC of 100 (testing accuracy 0.69, *P* < 0.0001). A 4-locus model consisted of those 3 SNPs in the above 3-locus model and an additional *SFRP1* rs16890282. However, this 4-locus model had a lower CVC of 94 and a lower testing accuracy of 0.63 than that in the 3-locus model (Table 5).

## Discussion

This is the first study to evaluate germline genetic variation in Wnt signaling pathway genes and risk of advanced hepatic fibrosis and inflammation in chronically HCV-infected males. We identified eight *SNPs* (*SFRP2* rs11937424, *SFRP2* rs7673508*, SFRP2* rs6853435, *SFRP2* rs3810765, *FZD4* rs11234870, *SOST* rs1405952, *CTNNB1* rs1798796 and *CSNK1A1* rs2431718) as predictors of advanced FibroSURE-determined fibrosis risk (F3/F4-F4 vs. F0-F3) in HCV+ males, and seven SNPs (*FZD1* rs1476442 and rs1346665, *SFRP1* rs16890282, *FZD8* rs3904594 and rs7920455, *TBX3* rs1386037 and rs1520177) as predictors of risk of advanced inflammation risk (A2/A3-A3 vs. A0-A2). 

A major finding in this study was the consistent allelic, genotypic and haplotypic associations between *SFRP2* rs11937424 and advanced fibrosis risk in HCV+ males. In single-locus association analysis, *SFRP2* rs11937424 had the strongest allelic (*P* = 0.0001) and genotypic association (*P* = 0.00004) with risk of advanced fibrosis. It was the only noteworthy SNP at the *a priori* specified FPRP low prior probability of 0.01. Haplotype analysis revealed that of the four observed haplotypes, the most significant risk haplotypes “A A A A” also carried the adverse *SFRP2* rs11937424 alleles (*P* = 0.0003). MDR analysis further confirmed the main effect of this SNP. It was the strongest one-locus model and was also part of the best 4-locus interaction model for predicting fibrosis risk. Thus, this significant association appeared to be consistent across all analyses, which strongly suggests that *SFRP2* rs11937424 or a genetic variant in LD with this SNP is associated with advanced HCV-related fibrosis risk in males. 

Although the functional relevance of individual *SFRP2* SNPs is unknown, several lines of evidence suggest that our epidemiologic findings are biologically plausible. As a major gene involved in Wnt signaling pathway, *SFRP2* (Secreted frizzled related protein 2, OMIM # 604157), is a key Frizzled regulator associated with a wide range of developmental and disease processes such as cancer, inflammation and fibrosis. One study reported that blocking *SFRP2* markedly reduces the extent and severity of fibrosis in mice with experimentally-induced myocardial infarction [25]. Another study also indicated a potential therapeutic application for *SFRP2* antagonism in controlling fibrosis in the infarcted heart [26]. Therefore, the biological functions of the SNPs in *SFRP2* identified in our study may warrant further investigation in studies of liver disease risk and progression.

Other interesting findings in this study are the association of *FZD1* and *TBX3* genes polymorphisms and advanced inflammation risk. *FZD1* has recently been implicated in the modulation of inflammatory processes [27].*TBX3* is a downstream target of the Wnt signaling pathway and a critical mediator of Wnt activities on cell proliferation and survival [28], with *TBX3* overexpression well-documented in liver tumorigenesis [29]. The association of *SOST* polymorphism and fibrosis risk is also of interest. The *SOST* product, sclerostin, is secreted by osteocytes and transported to the bone surface where it inhibits osteoblastic bone formation by antagonizing Wnt signaling. Serum sclerostin levels have recently been demonstrated to be significantly elevated in individuals with chronic alcohol abuse where they also correlated with decreased measures of bone turnover [30]. Given the high prevalence of chronic alcohol abuse in our cohort, additional research in larger HCV-infected cohorts is needed to elucidate whether the observed association with SOST and advanced fibrosis risk applies equally to patients with and without a history of alcohol abuse. 

**Table 5:** Summary of Multifactor Dimensionality Reduction (MDR) results for interactions analysis on advanced hepatic fibrosis and inflammation risk.

No. factor			Best candidate model		Testing accuracy	*P*-value #	OR (95% CI)	CVC *		
**Advanced hepatic fibrosis risk (F3/F4-F4 vs. F0-F3)**											
1	*SFRP2* rs11937424				0.62	< 0.0001	2.21 (1.48-3.30)		85/100	
2	*SOST* rs1405952, *CSNK1A1* rs2431718				0.60	< 0.0001	2.83 (1.86-4.31)		80/100	
3	*SOST* rs1405952, *SFRP2* rs11937424, *FZD4* rs11234870				0.65	< 0.0001	3.74 (2.43-5.73)		96/100	
4	Overweight/obese, *SOST* rs1405952, *SFRP2* rs11937424, *FZD4* rs11234870				**0.68**	< 0.0001	4.51 (2.94-6.91)		**97/100**	
5	Overweight/obese, *SOST* rs1405952, *SFRP2* rs11937424, *SFRP2* rs7673508, *FZD4* rs11234870				0.63	< 0.0001	5.86 (3.76-9.13)		89/100	
**Advanced hepatic inflammation risk (A2/A3-A3 vs. A0-A2)**											
1		*SFRP1* rs16890282			0.52	0.0004	2.11 (1.39-3.18)			64/100
2		*FZD1* rs1346665, *TBX3* rs1520177			0.51	< 0.0001	2.65 (1.78-3.93)			55/100
3		Race/Ethnicity, *FZD1* rs1346665, *TBX3* rs1520177			**0.69**	< 0.0001	3.43 (2.26-4.97)			**100/100**
4		Race/Ethnicity, *SFRP1* rs16890282, *FZD1* rs1346665, TBX3 rs1520177			0.63	< 0.0001	4.28 (2.85-6.42)			94/100
5		Race/Ethnicity, overweight/obese, *SFRP1* rs16890282, *FZD1* rs1476442, *TBX3* rs1520177			0.53	< 0.0001	5.08 (3.35,9.81)			72/100

^#^ *P*-value based on 10,000 permutations.

* Cross-validation Consistency.

Our study has several strengths. It was performed in a single large and well-phenotyped cohort with extensive data on potential confounding factors for advanced liver disease including obesity, alcohol abuse, and diabetes. A second strength was the relatively large sample size that provided sufficient statistical power for several findings. For example, we had 96% power to identify *SFRP2* rs1547711186 for risk of fibrosis (155 advanced and 270 mild fibrosis patients), based on the SNP have MAF of 0.28 and confer OR of 2.18 in a dominant model; we had 97% power to identify *SFRP1* rs16890282 associated with risk of inflammation (180 advanced and 245 mild inflammation patients), based on the variant having an MAF of 0.18 and conferring an OR of 2.15 in a dominant model. Nevertheless, further larger scale studies are warranted to validate our findings. Third strength is the use of the MDR method to identify higher-order interactions between clinical factors and genetic variants. It is well known that interactions are difficult to detect by traditional parametric statistical methods because of the sparseness of the data in higher dimensions. To address this problem, we adopted the MDR method for collapsing high-dimensional genotype predictors (i.e., SNPs) into a single dimension, thus permitting interactions to be detected in studies with relatively small sample sizes [20-22]. As our results showed, the interaction of overweight/obese, S*OST* rs1405952, *SFRP2* rs11937424 and *FZD4* rs11234870 was the best model for predicting risk of advanced fibrosis; whereas the interaction of race/ethnicity, *FZD1* rs1346665 and *TBX3* rs1520177 was the best model for predicting risk of advanced inflammation. 

Despite the strong association of certain SNPs with advanced FibroSURE-determined hepatic pathology, and the biologic plausibility for these associations, there were limitations of our study that need to be considered when drawing inferences. First, our study was cross-sectional in design. Although this prohibits drawing a causal inference, this is of less concern in our genetic association study where germline genotype by definition predates adult-acquired HCV-related liver disease phenotype. Second, there could be discordance between liver biopsy, and FibroSURE testing. Although liver biopsy is still considered the ‘gold standard’, it is well-known to be subject to variability due to sampling and pathologist interpretation. Our use of the FibroSURE test which was uniformly obtained on all patients recruited in this study reduces the considerable degree of selection bias that would have otherwise been introduced if we relied on liver biopsy which is typically performed only in small minority of patients eligible for treatment. Further, the FibroSURE-ActiTest has been well-validated with good performance characteristics in multiple HCV populations [14-16]. A third limitation is our absence of a parallel measure of gene function and the unknown functional significance of some of individual SNPs identified as significant in our analyses. Another limitation is our study population included only HCV+ men. Several recent experimental studies have demonstrated substantial interaction between the canonical Wnt-beta catenin signaling pathway and the androgen receptor or androgens, including androgen activation of beta-catenin signaling in bladder cancer cells [31] and improved liver histology from transplantation therapy with bone marrow derived mesenchymal stem cells in a murine liver cirrhosis model with use of therapies targeting the androgen receptor [32]. This experimental data in conjunction with the well-known lower risk of advanced fibrosis in females suggests caution in generalizing our findings to HCV+ women pending performance of additional adequately powered gender-specific research. Finally, we also had a limited sample size of African American males limiting our power in race-stratified analyses and had insufficient power to use other genetic markers to adjust for potential admixture. Notably, many of the associations that were significant in overall analyses and in race-stratified analyses in Whites were similar in direction and magnitude in African Americans, even though non-significant. However, additional studies in larger HCV+ cohorts will be needed to more precisely define the magnitude of associations between these individual Wnt pathway SNPs and advanced liver disease risk in African American males. 

In summary, our findings suggest germline polymorphisms in several Wnt signaling genes are associated with risk of advanced hepatic fibrosis or inflammation in males with chronic HCV. The Wnt pathway is increasingly recognized as critical in driving inflammatory response and carcinogenesis, including hepatocarcinogenesis. Our findings of increased risk of advanced fibrosis, the precursor lesion for most cases of liver cancer, with SNPs in several Wnt pathway genes raises the intriguing possibility that specific Wnt antagonists or inverse agonists could have beneficial effects in treatment or chemoprevention for HCV-related liver disease in males. Additional research is warranted to confirm these findings in other HCV-infect male populations and to ascertain the role of Wnt pathway genes in HCV-related disease progression in females. 

## Supporting Information

Table S1 **The list of Wnt signaling pathway genes evaluated in the present study.**
(DOCX)

Table S2 **Genotype frequencies of SNPs and the associations with advanced hepatic fibrosis or inflammation risk (0.001 < P ≤ 0.01).**
(DOCX)

Table S3 **Logistic regression analysis of the main effect SNPs genotypes stratified by race/ethnicity.**
(DOCX)

Table S4 **The measure of LD (*D*’ and **r**) among all possible pairs of SNPs.**
(DOCX)

## References

[1] World Health Organization (2004) Global burden of disease (GBD) for hepatitis C. J Clin Pharmacol 44: 20–29. doi:10.1177/0091270003258669. PubMed: 14681338. 14681338

[2] Armstrong GL, Wasley A, Simard EP, McQuillan GM, Kuhnert WL et al. (2006) The prevalence of hepatitis C virus infection in the United States, 1999 through 2002. Ann Intern Med 144: 705-714. doi:10.7326/0003-4819-144-10-200605160-00004. PubMed: 16702586. 16702586

[3] Davila JA, Morgan RO, Shaib Y, McGlynn KA, El-Serag HB (2004) Hepatitis C infection and the increasing incidence of hepatocellular carcinoma: a population-based study. Gastroenterology 127: 1372-1380. doi:10.1053/j.gastro.2004.07.020. PubMed: 15521006. 15521006

[4] Monga SP, Monga HK, Tan X, Mulé K, Pediaditakis P et al. (2003) Beta-catenin antisense studies in embryonic liver cultures: role in proliferation, apoptosis, and lineage specification. Gastroenterology 124: 202-216. doi:10.1016/S0016-5085(03)81011-9. PubMed: 12512043. 12512043

[5] Micsenyi A, Tan X, Sneddon T, Luo JH, Michalopoulos GK et al. (2004) Beta-catenin is temporally regulated during normal liver development. Gastroenterology 126: 1134-1146. doi:10.1053/j.gastro.2003.12.047. PubMed: 15057752. 15057752

[6] Cadoret A, Ovejero C, Terris B, Souil E, Lévy L et al. (2002) New targets of beta-catenin signaling in the liver are involved in the glutamine metabolism. Oncogene 21: 8293-8301. doi:10.1038/sj.onc.1206118. PubMed: 12447692. 12447692

[7] Sekine S, Lan BY, Bedolli M, Feng S, Hebrok M (2006) Liver-specific loss of beta-catenin blocks glutamine synthesis pathway activity and cytochrome p450 expression in mice. Hepatology 43: 817-825. doi:10.1002/hep.21131. PubMed: 16557553. 16557553

[8] Thorgeirsson SS, Grisham JW (2002) Molecular pathogenesis of human hepatocellular carcinoma. Nat Genet 31: 339-346. doi:10.1038/ng0802-339. PubMed: 12149612. 12149612

[9] Takahara Y, Takahashi M Zhan QW, Wagatsuma H, Mori M et al. (2008) Serial changes in expression of functionally clustered genes in progression of liver fibrosis. World J Gastroenterol 14(13): 2010-2022. doi:10.3748/wjg.14.2010. PubMed: 18395900. 18395900 PMC2701521

[10] Merle P, Kim M, Herrmann M, Gupte A, Lefrançois L et al. (2005) Oncogenic role of the frizzled-7/beta-catenin pathway in hepatocellular carcinoma. J Hepatol 43: 854-862. doi:10.1016/j.jhep.2005.05.018. PubMed: 16098625. 16098625

[11] Buendia MA (2000) Genetics of hepatocellular carcinoma. Semin Cancer Biol 10: 185-200. doi:10.1006/scbi.2000.0319. PubMed: 10936068. 10936068

[12] White DL, Tavakoli-Tabasi S, Kuzniarek J, Pascua R, Ramsey DJ et al. (2012) Higher serum testosterone is associated with increased risk of advanced hepatitis C-related liver disease in males. Hepatology 55: 759-768. doi:10.1002/hep.24618. PubMed: 21858849. 21858849 PMC3399504

[13] Halfon P, Bourliere M, Deydier R, Botta-Fridlund D, Renou C et al. (2006) Independent prospective multicenter validation of biochemical markers (fibrotest-actitest) for the prediction of liver fibrosis and activity in patients with chronic hepatitis C: the fibropaca study. Am J Gastroenterol 101: 547-555. doi:10.1111/j.1572-0241.2006.00411.x. PubMed: 16542291. 16542291

[14] Poynard T, de Ledinghen V, Zarski JP, Stanciu C, Munteanu M et al. (2012) Relative performances of FibroTest, Fibroscan, and biopsy for the assessment of the stage of liver fibrosis in patients with chronic hepatitis C: a step toward the truth in the absence of a gold standard. J Hepatol 56: 541-548. doi:10.1016/S0168-8278(12)61388-9. PubMed: 21889468. 21889468

[15] Patel K, Benhamou Y, Yoshida EM, Kaita KD, Zeuzem S et al. (2009) An independent and prospective comparison of two commercial fibrosis marker panels (HCV FibroSURE and FIBROSpect II) during albinterferon alfa-2b combination therapy for chronic hepatitis C. J Viral Hepat 16: 178-186. doi:10.1111/j.1365-2893.2008.01062.x. PubMed: 19175870. 19175870

[16] Poynard T, Munteanu M, Ngo Y, Castera L, Halfon P et al. (2010) ActiTest accuracy for the assessment of histological activity grades in patients with chronic hepatitis C, an overview using Obuchowski measure. Gastroenterol Clin Biol 34: 388-396. doi:10.1016/j.gcb.2010.05.001. PubMed: 20580175. 20580175

[17] Poynard T, Imbert-Bismut F, Ratziu V, Chevret S, Jardel C et al. (2002) Biochemical markers of liver fibrosis in patients infected by hepatitis C virus: longitudinal validation in a randomized trial. J Viral Hepat 9: 128-133. doi:10.1046/j.1365-2893.2002.00341.x. PubMed: 11876795. 11876795

[18] Akaike H (1974) A new look at the statistical model identification. IEEE Trans. Automat Contr 19: 716-723. doi:10.1109/TAC.1974.1100705.

[19] Wacholder S, Chanock S, Garcia-Closas M, El Ghormli L, Rothman N (2004) Assessing the probability that a positive report is false: an approach for molecular epidemiology studies. J Natl Cancer Inst 96: 434-442. doi:10.1093/jnci/djh075. PubMed: 15026468. 15026468 PMC7713993

[20] Ritchie MD, Hahn LW, Roodi N, Bailey LR, Dupont WD et al. (2001) Multifactor-dimensionality reduction reveals high-order interactions among estrogen-metabolism genes in sporadic breast cancer. Am J Hum Genet 69: 138-147. doi:10.1086/321276. PubMed: 11404819. 11404819 PMC1226028

[21] Moore JH, Williams SM (2002) New strategies for identifying gene-gene interactions in hypertension. Ann Med 34: 88-95. doi:10.1080/07853890252953473. PubMed: 12108579. 12108579

[22] Hahn LW, Ritchie MD, Moore JH (2003) Multifactor dimensionality reduction software for detecting gene-gene and gene-environment interactions. Bioinformatics 19: 376-382. doi:10.1093/bioinformatics/btf869. PubMed: 12584123. 12584123

[23] Lewontin RC (1988) On Measures of Gametic Disequilibrium. Genetics 120: 849–852. PubMed: 3224810. 3224810 10.1093/genetics/120.3.849 PMC1203562

[24] Schaid DJ, Rowland CM, Tines DE, Jacobson RM, Poland GA (2002) Score tests for association between traits and haplotypes when linkage phase is ambiguous. Am J Hum Genet 70: 425-434. doi:10.1086/338688. PubMed: 11791212. 11791212 PMC384917

[25] He W, Zhang L, Ni A, Zhang Z, Mirotsou M et al. (2010) Exogenously administered secreted frizzled related protein 2 (Sfrp2) reduces fibrosis and improves cardiac function in a rat model of myocardial infarction. Proc Natl Acad Sci U S A 107: 21110-21115. doi:10.1073/pnas.1004708107. PubMed: 21078975. 21078975 PMC3000293

[26] Kobayashi K, Luo M, Zhang Y, Wilkes DC, Ge G et al. (2009) Secreted Frizzled-related protein 2 is a procollagen C proteinase enhancer with a role in fibrosis associated with myocardial infarction. Nat Cell Biol 11: 46-55. doi:10.1038/ncb1811. PubMed: 19079247. 19079247 PMC2722759

[27] Neumann J, Schaale K, Farhat K, Endermann T, Ulmer AJ et al. (2010) Frizzled1 is a marker of inflammatory macrophages, and its ligand Wnt3a is involved in reprogramming Mycobacterium tuberculosis-infected macrophages. FASEB J 24: 4599-4612. doi:10.1096/fj.10-160994. PubMed: 20667980. 20667980

[28] Renard CA, Labalette C, Armengol C, Cougot D, Wei Y et al. (2007) Tbx3 is a downstream target of the Wnt/beta-catenin pathway and a critical mediator of beta-catenin survival functions in liver cancer. Cancer Res 67: 901-910. doi:10.1158/0008-5472.CAN-06-2344. PubMed: 17283120. 17283120

[29] Suzuki A, Sekiya S, Büscher D, Izpisúa Belmonte JC, Taniguchi H (2008) Tbx3 controls the fate of hepatic progenitor cells in liver development by suppressing p19ARF expression. Development 135: 1589-1595. doi:10.1242/dev.016634. PubMed: 18356246. 18356246

[30] González-Reimers E, Martín-González C, de la Vega-Prieto MJ, Pelazas-González R, Fernández-Rodríguez C et al. (2013) Serum sclerostin in alcoholics: a pilot study. Alcohol Alcohol 48: 278-282. doi:10.1093/alcalc/ags136. PubMed: 23296214. 23296214

[31] Li Y, Zheng Y, Izumi K, Ishiguro H, Ye B et al. (2013) Androgen a ctivates B-catenin signaling in bladder cancer cells. Endocr Relat Cancer 20(3): 293-304. doi:10.1530/ERC-12-0328. PubMed: 23447569. 23447569

[32] Huang C-K, Lee SO, Lai K-P, Ma WL, Lin TH et al. (2013) Targeting androgen receptor in bone marrow mesenchymal stem cells leads to better transplantation therapy efficacy in liver cirrhosis. Hepatology 57: 1550-1563. doi:10.1002/hep.26135. PubMed: 23150236. 23150236

