# Supplementary material for: WNT Signaling Pathway Gene Polymorphisms and Risk of Hepatic Fibrosis and Inflammation in HCV-Infected Patients
Source: PLoS One. 2013 Dec 30;8(12):e84407. doi: 10.1371/journal.pone.0084407 (PMC3875538; doi:10.1371/journal.pone.0084407)
Supplement: Table S1 — The list of Wnt signaling pathway genes evaluated in the present study. (DOCX) [file pone.0084407.s001.docx]

**Table S1. The list of Wnt signaling pathway genes evaluated in the present study**

| **Family** | **Function** | **Genes** |
| --- | --- | --- |
| WNT | ligand | *WNT1, WNT2, WNT2B, WNT3, WNT3A, WNT4, WNT5A, WNT5B, WNT6, WNT7A, WNT7B, WNT8A, WNT8B, WNT9A, WNT9B, WNT10A, WNT10B, WNT11, WNT16* |
| Frizzled | receptor | *FZD1, FZD2, FZD3, FZD4, FZD5, FZD6, FZD7, FZD8, FZD9, FZD10, FZD11* |
| SFRP | Wnt antagonist | *SFRP1, SFRP2, FRZB, SFRP4, SFRP5, DKK1, DKK2, DKK3, DKK4, WIF1, SOST* |
| LRP | receptor | *LRP5, LRP6* |
| APC | regulator | *ANAPC1, APC2* |
| Dishevelled | regulator | *DVL1, DVL2, DVL3, DVL1L1* |
| AXIN | regulator | *AXIN1, AXIN2* |
| GSK3 | regulator | *GSK3A, GSK3B* |
| WTX | regulator | *FAM123B* |
| CK | regulator | *CSNK1A1* |
| TCF | transcription | *TCF7L2* |
| Beta-catenin | transcription | *CTNNB1* |
| TBX | target | *TBX3* |
