# Supplementary material for: WNT Signaling Pathway Gene Polymorphisms and Risk of Hepatic Fibrosis and Inflammation in HCV-Infected Patients
Source: PLoS One. 2013 Dec 30;8(12):e84407. doi: 10.1371/journal.pone.0084407 (PMC3875538; doi:10.1371/journal.pone.0084407)
Supplement: Table S2 — Genotype frequencies of SNPs and the associations with advanced hepatic fibrosis or inflammation risk (0.001 < P ≤ 0.01). (DOCX) [file pone.0084407.s002.docx]

**Table S2.** Genotype frequencies of SNPs and the associations with advanced hepatic fibrosis or inflammation risk (0.001 < P ≤ 0.01)

| Chr | Gene | RS ID | Model ^ǂ^ | Coordinate | Minor.Allele | MAF.total | Logistic regression* | | | |
| --- | --- | --- | --- | --- | --- | --- | --- | --- | --- | --- |
|  |  |  |  |  |  |  | OR | 95% CI | | *P-*value |
| **Advanced hepatic fibrosis risk (F3/F4-F4 vs. F0-F3)** | | | | |  |  |  |  |  |  |
| 1 | *WNT4* | rs10917196 | d | 22622513 | A | 0.43 | 1.95 | 1.24 | 3.08 | 0.0039 |
| 3 | *CTNNB1* | rs6788984 | d | 41107173 | G | 0.19 | 1.94 | 1.25 | 3.04 | 0.0035 |
| 3 | *CTNNB1* | rs11716816 | r | 41037617 | G | 0.44 | 0.46 | 0.27 | 0.78 | 0.0038 |
| 3 | *CTNNB1* | rs6798084 | d | 41032309 | G | 0.20 | 1.74 | 1.14 | 2.66 | 0.0100 |
| 3 | *CTNNB1* | rs6768379 | r | 40904201 | A | 0.27 | 2.41 | 1.23 | 4.75 | 0.0107 |
| 3 | *DVL3* | rs16858473 | r | 183867622 | A | 0.06 | 0.52 | 0.32 | 0.84 | 0.0084 |
| 3 | *WNT5A* | rs4955830 | d | 55179991 | G | 0.07 | 0.38 | 0.19 | 0.77 | 0.0073 |
| 3 | *WNT7A* | rs12634112 | d | 13878889 | A | 0.16 | 0.47 | 0.29 | 0.78 | 0.0029 |
| 4 | *DKK2* | rs7672970 | r | 107470497 | G | 0.28 | 0.23 | 0.08 | 0.61 | 0.0033 |
| 4 | *DKK2* | rs10019755 | r | 108181819 | G | 0.45 | 0.49 | 0.29 | 0.83 | 0.0080 |
| 4 | *SFRP2* | rs5009912 | d | 154791497 | A | 0.49 | 2.06 | 1.23 | 3.46 | 0.0063 |
| 4 | *SFRP2* | rs11723258 | r | 154733070 | G | 0.44 | 0.46 | 0.26 | 0.81 | 0.0074 |
| 4 | *SFRP2* | rs4696523 | d | 154884393 | A | 0.08 | 0.41 | 0.21 | 0.79 | 0.0080 |
| 4 | *SFRP2* | rs10031057 | d | 154746806 | G | 0.45 | 1.91 | 1.18 | 3.09 | 0.0087 |
| 4 | *SFRP2* | rs4696512 | r | 154773027 | G | 0.48 | 0.49 | 0.28 | 0.84 | 0.0104 |
| 7 | *FZD1* | rs1346665 | r | 90919567 | A | 0.47 | 0.47 | 0.28 | 0.80 | 0.0055 |
| 8 | *SFRP1* | rs6983729 | d | 41028520 | A | 0.16 | 0.50 | 0.31 | 0.81 | 0.0049 |
| 11 | *DKK3* | rs7396140 | d | 11996757 | G | 0.35 | 1.78 | 1.15 | 2.73 | 0.0089 |
| 11 | *WNT11* | rs11236683 | r | 75996547 | G | 0.31 | 2.75 | 1.42 | 5.32 | 0.0026 |
| 11 | *WNT11* | rs4944098 | r | 75964525 | A | 0.28 | 2.50 | 1.25 | 5.03 | 0.0098 |
| 12 | *FZD10* | rs10848029 | d | 130665452 | A | 0.08 | 0.44 | 0.24 | 0.81 | 0.0089 |
| 12 | *TBX3* | rs254694 | r | 115589524 | G | 0.48 | 2.21 | 1.32 | 3.70 | 0.0025 |
| 12 | *TBX3* | rs7304809 | d | 116244393 | G | 0.16 | 2.59 | 1.33 | 5.03 | 0.0050 |
| 12 | *TBX3* | rs7976017 | r | 115638790 | G | 0.14 | 9.42 | 1.90 | 46.79 | 0.0061 |
| 12 | *TBX3* | rs6490040 | d | 116248048 | A | 0.15 | 2.08 | 1.21 | 3.57 | 0.0079 |
| 17 | *AXIN2* | rs985154 | r | 63326210 | A | 0.23 | 4.42 | 1.58 | 12.36 | 0.0046 |
| 17 | *WNT3* | rs11079737 | r | 44888209 | A | 0.23 | 0.21 | 0.07 | 0.64 | 0.0059 |
| **Advanced hepatic inflammation risk (A2/A3-A3 vs. A0-A2)** | | | | | |  |  |  |  |  |
| 1 | *WNT2B* | rs351354 | d | 113038795 | A | 0.16 | 1.78 | 1.15 | 2.74 | 0.0091 |
| 1 | *WNT4* | rs2744752 | r | 22575306 | A | 0.07 | 0.46 | 0.29 | 0.74 | 0.0014 |
| 2 | *FZD7* | rs10931978 | r | 202775055 | G | 0.42 | 0.47 | 0.27 | 0.82 | 0.0075 |
| 3 | *DVL3* | rs16858473 | r | 183867622 | A | 0.06 | 0.45 | 0.28 | 0.73 | 0.0012 |
| 3 | *WNT5A* | rs13083875 | r | 55322453 | A | 0.05 | 0.46 | 0.29 | 0.74 | 0.0012 |
| 3 | *WNT5A* | rs2004213 | r | 55333096 | A | 0.05 | 0.46 | 0.29 | 0.74 | 0.0013 |
| 3 | *WNT7A* | rs13097641 | r | 13825666 | G | 0.41 | 2.29 | 1.31 | 3.98 | 0.0034 |
| 4 | *DKK2* | rs9995574 | r | 107855336 | G | 0.07 | 0.46 | 0.29 | 0.74 | 0.0012 |
| 4 | *DKK2* | rs6849760 | d | 108007908 | A | 0.06 | 0.39 | 0.20 | 0.76 | 0.0062 |
| 4 | *SFRP2* | rs7661078 | r | 154883600 | A | 0.28 | 0.27 | 0.10 | 0.69 | 0.0065 |
| 7 | *FZD1* | rs12155087 | r | 91314594 | G | 0.06 | 0.46 | 0.29 | 0.74 | 0.0012 |
| 7 | *FZD1* | rs9785984 | r | 91108429 | A | 0.36 | 0.42 | 0.26 | 0.69 | 0.0067 |
| 7 | *FZD1* | rs992769 | r | 91210770 | G | 0.49 | 0.52 | 0.33 | 0.85 | 0.0080 |
| 7 | *FZD1* | rs2540592 | r | 91233469 | G | 0.50 | 0.54 | 0.34 | 0.86 | 0.0089 |
| 8 | *SFRP1* | rs17571033 | r | 40810322 | G | 0.06 | 0.46 | 0.29 | 0.74 | 0.0012 |
| 8 | *SFRP1* | rs7813807 | r | 40914727 | A | 0.48 | 1.87 | 1.18 | 2.98 | 0.0079 |
| 10 | *DKK1* | rs12412036 | r | 54294077 | A | 0.05 | 0.46 | 0.29 | 0.74 | 0.0012 |
| 10 | *DKK1* | rs10824669 | r | 54372399 | A | 0.47 | 2.01 | 1.25 | 3.23 | 0.0037 |
| 10 | *DKK1* | rs2891402 | r | 54505972 | A | 0.25 | 3.44 | 1.41 | 8.44 | 0.0068 |
| 10 | *FZD8* | rs17638209 | r | 36993200 | A | 0.06 | 0.46 | 0.29 | 0.74 | 0.0012 |
| 10 | *FZD8* | rs1914170 | r | 37342776 | A | 0.06 | 0.46 | 0.29 | 0.74 | 0.0012 |
| 10 | *FZD8* | rs11010839 | r | 37048760 | A | 0.06 | 0.46 | 0.29 | 0.74 | 0.0014 |
| 10 | *FZD8* | rs16937124 | r | 36885746 | C | 0.05 | 0.47 | 0.30 | 0.76 | 0.0021 |
| 10 | *FZD8* | rs10508828 | d | 36344376 | A | 0.13 | 0.51 | 0.31 | 0.83 | 0.0068 |
| 10 | *FZD8* | rs618443 | d | 35948710 | A | 0.18 | 1.94 | 1.20 | 3.15 | 0.0070 |
| 10 | *FZD8* | rs1192658 | d | 37158006 | A | 0.07 | 0.43 | 0.23 | 0.81 | 0.0088 |
| 10 | *FZD8* | rs3851074 | r | 36337678 | A | 0.49 | 1.85 | 1.16 | 2.95 | 0.0092 |
| 10 | *FZD8* | rs12782605 | r | 36346582 | A | 0.47 | 1.90 | 1.17 | 3.09 | 0.0094 |
| 10 | *FZD8* | rs2503044 | d | 36734783 | A | 0.25 | 0.57 | 0.37 | 0.87 | 0.0099 |
| 11 | *DKK3* | rs12421658 | r | 12016945 | A | 0.07 | 0.46 | 0.29 | 0.74 | 0.0012 |
| 12 | *FZD10* | rs12827481 | r | 130692074 | A | 0.06 | 0.46 | 0.29 | 0.74 | 0.0012 |
| 12 | *FZD10* | rs12822055 | r | 130678432 | G | 0.06 | 0.48 | 0.30 | 0.77 | 0.0023 |
| 12 | *FZD10* | rs11616174 | d | 130814503 | G | 0.30 | 0.54 | 0.34 | 0.83 | 0.0055 |
| 12 | *TBX3* | rs7962752 | r | 115995618 | G | 0.06 | 0.46 | 0.29 | 0.74 | 0.0012 |
| 12 | *TBX3* | rs1896350 | r | 115382425 | A | 0.05 | 0.46 | 0.29 | 0.74 | 0.0012 |
| 12 | *TBX3* | rs2062717 | r | 115174093 | A | 0.45 | 1.88 | 1.17 | 3.02 | 0.0092 |
| 12 | *WIF1* | rs11175619 | r | 65391905 | A | 0.18 | 6.36 | 1.74 | 23.22 | 0.0051 |
| 12 | *WNT5B* | rs3803163 | r | 1755960 | A | 0.07 | 0.46 | 0.29 | 0.74 | 0.0012 |
| 17 | *AXIN2* | rs8076281 | r | 63332896 | G | 0.18 | 5.60 | 1.71 | 18.38 | 0.0045 |
| 17 | *AXIN2* | rs985574 | r | 63329765 | G | 0.20 | 3.75 | 1.45 | 9.66 | 0.0062 |
| 17 | *SOST* | rs1881107 | r | 41786076 | G | 0.50 | 0.53 | 0.33 | 0.86 | 0.0095 |

NOTE: MAF, minor allele frequency; OR, odds ratio; CI, confidence interval.

^ǂ^ The Akaike's information criterion was used to determine the genetic model for each SNP. D, dominant; R, recessive.

* Adjusted for age, race/ethnicity, presence of overweight/obesity (BMI > 25), chronic alcohol abuse, diabetes, viral load, and HCV genotypes.
