# Supplementary material for: WNT Signaling Pathway Gene Polymorphisms and Risk of Hepatic Fibrosis and Inflammation in HCV-Infected Patients
Source: PLoS One. 2013 Dec 30;8(12):e84407. doi: 10.1371/journal.pone.0084407 (PMC3875538; doi:10.1371/journal.pone.0084407)
Supplement: Table S3 — Logistic regression analysis of the main effect SNPs genotypes stratified by race/ethnicity. (DOCX) [file pone.0084407.s003.docx]

**Table S3.** Logistic regression analysis of the main effect SNPs genotypes stratified by race/ethnicity

| SNP^ǂ^ | Whites (n=313) |  | |  | African Americans (n=112) |  |
| --- | --- | --- | --- | --- | --- | --- |
|  | OR (95% CI) * | *P*-value* | |  | OR (95% CI) * | *P*-value* |
| **Advanced hepatic fibrosis risk (F3/F4-F4 vs. F0-F3)** | | | |  |  |  |
| rs1798796 ^r^ | 0.38 (0.14 - 0.85) | 0.036 | |  | 1.06 (0.39 - 2.86) | 0.91 |
| rs6853435 ^r^ | 0.49 (0.28 - 0.87) | 0.012 | |  | 0.52 (0.18 - 1.52) | 0.21 |
| rs3810765 ^r^ | 0.49 (0.29 - 0.86) | 0.009 | |  | 0.56 (0.19 - 1.64) | 0.27 |
| rs11937424 ^d^ | 2.04 (1.36 - 3.21) | 0.0006 | |  | 2.33 (0.85 - 6.25) | 0.10 |
| rs7673508 ^d^ | 1.89 (1.11 - 3.13) | 0.016 | |  | 1.49 (0.64 - 3.45) | 0.36 |
| rs2431718 ^d^ | 0.45 (0.28 - 0.72) | 0.0007 | |  | 1.47 (0.58 - 3.70) | 0.41 |
| rs11234870 ^d^ | 2.22 (1.39 - 3.57) | 0.0007 | |  | 1.85 (0.82 - 4.17) | 0.14 |
| rs1405952 ^d^ | 2.38 (1.47 - 3.85) | 0.0004 | |  | 2.04 (0.88 - 4.76) | 0.10 |
| **Advanced hepatic inflammation risk (A2/A3-A3 vs. A0-A2)** | | | |  |  |  |
| rs1346665 ^r^ | 0.51 (0.29 - 0.92) | | 0.022 |  | 0.93 (0.35 - 2.50) | 0.89 |
| rs1476442 ^d^ | 2.22 (1.35 - 3.57) | | 0.0013 |  | 1.56 (0.64 - 3.85) | 0.32 |
| rs16890282 ^d^ | 1.72 (1.09 - 2.70) | | 0.021 |  | 3.13 (0.88 - 11.11) | 0.078 |
| rs3904594 ^r^ | 0.53 (0.33 - 0.85) | | 0.0091 |  | 0.64 (0.28 - 1.47) | 0.28 |
| rs7920455 ^d^ | 0.54 (0.34 - 0.84) | | 0.0064 |  | 0.64 (0.28 - 1.47) | 0.30 |
| rs1520177 ^d^ | 1.59 (1.02 - 2.50) | | 0.041 |  | 1.64 (0.63 - 4.35) | 0.31 |
| rs1346665 ^r^ | 0.49 (0.30 - 0.81) | | 0.0044 |  | 0.51 (0.20 - 1.27) | 0.15 |

NOTE: OR, odds ratio; CI, confidence interval.

^ǂ^ The Akaike's information criterion was used to determine the genetic model for each SNP. D, dominant; R, recessive.

* Adjusted for age, ethnicity, presence of overweight obesity (BMI > 25), chronic alcohol abuse, diabetes, viral load, and HCV genotypes.
