# Supplementary material for: WNT Signaling Pathway Gene Polymorphisms and Risk of Hepatic Fibrosis and Inflammation in HCV-Infected Patients
Source: PLoS One. 2013 Dec 30;8(12):e84407. doi: 10.1371/journal.pone.0084407 (PMC3875538; doi:10.1371/journal.pone.0084407)
Supplement: Table S4 — The measure of LD (D’ and r) among all possible pairs of SNPs. (DOCX) [file pone.0084407.s004.docx]

**Table S4.** The measure of LD (*D’* and r) among all possible pairs of SNPs

| **Advanced hepatic fibrosis risk (F3/F4-F4 vs. F0-F3)** | | | | |
| --- | --- | --- | --- | --- |
| *SFRP2* | **D' (r)** | rs3810765 | rs11937424 | rs7673508 |
|  | rs6853435 | 0.99 (0.99) | 0.98 (-0.55) | 0.95 (-0.69) |
|  | rs3810765 |  | 0.98 (-0.55) | 0.95 (-0.69) |
|  | rs11937424 |  |  | 0.72 (0.56) |
| **Advanced hepatic inflammation risk (A2/A3-A3 vs. A0-A2)** | | | | |
| *FZD1* | **D' (r)** | rs1476442 |  |  |
|  | rs1346665 | 0.12 (0.03) |  |  |
| *FZD8* | **D' (r)** | rs3904594 |  |  |
|  | rs7920455 | 0.99 (-0.67) |  |  |
| *TBX3* | **D' (r)** | rs1520177 |  |  |
|  | rs1386037 | 0.07 (-0.04) |  |  |
